# Association of Senior Dance Participation with Mental Well-Being and Functional Performance in Older Women: A Cross-Sectional Study

**DOI:** 10.3390/jcm15197687

**Published:** 2026-10-04

**Authors:** Brigitta Lakó, András Simon, Anna Zsófia Kubik, Zsigmond Gyombolai, Éva Kovács

**Affiliations:** 1 Health Sciences Division, Doctoral College, Semmelweis University, 1085 Budapest, Hungary; arndt.brigitta@semmelweis.hu; 2 Department of Physiotherapy, Faculty of Health Sciences, Semmelweis University, 1085 Budapest, Hungary; simon.andras@semmelweis.hu (A.S.); kubik.anna@phd.semmelweis.hu (A.Z.K.); 3 Department of Morphology and Physiology, Faculty of Health Sciences, Semmelweis University, 1085 Budapest, Hungary; gyombolai.zsigmond@semmelweis.hu

**Keywords:** senior dance, older women, functional mobility, functional performance, functional aerobic endurance, mental well-being, physical activity, healthy aging

## Abstract

**Background/Objectives:** Community-based activities supporting physical function and mental well-being are increasingly relevant in later life. This cross-sectional study compared mental well-being and physical outcomes among older women participating in senior dance only, senior dance plus additional regular physical activity, and physically inactive older groups; a younger inactive group provided exploratory age-related context. A secondary aim was to examine correlations with participation duration. **Methods**: Participants were 160 women: 66 in senior dance only (SD), 34 in senior dance plus additional regular physical activity (SD+PA), 30 physically inactive older women, and 30 physically inactive younger women aged 25–40 years. Mental well-being was assessed with the Hungarian four-response five-item Well-Being Index (WBI-5), together with seven physical outcomes. Welch’s ANOVA and prespecified Welch–Satterthwaite contrasts with Benjamini–Hochberg correction were used; duration analyses used partial Spearman correlations adjusted for weekly attendance and age. **Results**: Overall group differences were observed for all outcomes (all *p* ≤ 0.002). Seven outcomes remained significant in Contrast 1, which did not isolate a dance-specific association. No SD versus SD+PA difference remained significant after correction. Within SD, participation duration correlated with more favorable Timed Up and Go (r_s_ = −0.428), 30-Second Chair Stand (r_s_ = 0.425), and 2-Minute Step Test performance (r_s_ = 0.423; all q = 0.0013). The adjusted WBI-5 correlation did not remain significant after FDR correction (r_s_ = 0.271; q = 0.061). **Conclusions**: Participation duration was most consistently correlated with integrated functional performance. Prospective studies are required; causal effects cannot be inferred. ClinicalTrials.gov: NCT07170241.

## 1. Introduction

Population aging represents a major demographic and public-health challenge worldwide. The United Nations projects that the global population aged 60 years and older will reach approximately 2.1 billion by 2050 [1].

Functional fitness encompasses the physical capacities required to perform activities of daily living safely, independently, and without excessive fatigue. Relevant capacities include muscle strength, functional aerobic endurance, flexibility, and integrated functional mobility. These capacities may decline with advancing age, whereas regular physical activity is associated with a lower risk of functional limitation and disability in older adults [2,3,4].

Dance is a multimodal form of physical activity that combines repetitive stepping, weight transfer, changes in direction, rhythmic coordination, motor learning, and social interaction. Systematic reviews and meta-analyses suggest that dance-based programs may favorably influence functional mobility, balance-related performance, gait, lower-limb function, and functional endurance in older adults [5,6,7,8].

However, the magnitude and consistency of these findings vary across dance modalities and outcomes, and evidence regarding isolated muscle strength, body composition, and flexibility is less consistent [6,7].

Senior dance is a structured, group-based, choreographed form of dance adapted to older participants. Published Senior Dance programs use rhythmic choreographies comprising relatively simple coordinated movements performed individually, in pairs, circles, or groups and are designed to challenge balance, motor coordination, and movement sequencing while remaining adaptable to older adults’ functional capacities [9,10]. The rationale for focusing on integrated functional performance is supported by intervention and review evidence indicating that dance-based programs in older adults can influence mobility, balance-related performance, lower-limb function, and functional endurance, although findings vary by dance modality, outcome, and comparator [5,6,7,8,9,10].

Most available evidence has been derived from structured, time-limited dance interventions, with substantial heterogeneity in program duration, frequency, content, delivery, and adherence [8,10]. Less is known about adults who participate in established community-based senior dance over extended periods or whether engagement in other regular physical activity alongside senior dance is associated with a different outcome profile. The contribution of the present study is therefore to characterize established community-based senior dance using a multidomain outcome profile, to distinguish participation duration from current weekly attendance frequency, and to examine whether additional structured physical activity is associated with different outcomes.

The primary aim of this study was to compare mental well-being and physical outcomes among older women participating exclusively in senior dance, older women combining senior dance with additional regular physical activity, and physically inactive older women. Comparisons involving physically inactive women aged 25–40 years were used to provide exploratory age-related context. A secondary aim was to examine correlations between senior dance participation duration and study outcomes within the senior dance-only group.

The group comparison hypotheses were evaluated using prespecified planned contrasts, as detailed in Section 2.6. Comparisons involving the younger reference group were regarded as exploratory and were not formulated as equivalence or non-inferiority hypotheses. For the duration analyses, we hypothesized that participation duration would correlate with more favorable mental well-being and physical-function outcomes after adjustment for current weekly senior dance attendance frequency and chronological age.

## 2. Materials and Methods

### 2.1. Study Design and Setting

This comparative cross-sectional study examined functional performance and mental well-being among women participating in senior dance and physically inactive comparison groups. Data were collected between September 2025 and March 2026 at Semmelweis University in Budapest, Hungary, and at the Idősek Közösségi Központja (Senior Center) in Szeged, Hungary. Reporting was guided by the Strengthening the Reporting of Observational Studies in Epidemiology (STROBE) recommendations for cross-sectional studies [11].

### 2.2. Participants and Recruitment

The primary analytical population comprised three groups of older women: participants attending senior dance only, participants combining senior dance with other regular physical activity, and physically inactive older controls. A physically inactive younger adult group was included as an exploratory reference group and was not used for primary hypothesis testing.

A total of 189 participants were recruited and completed the study assessments. The present analysis was restricted post hoc to women because the number of male participants within individual groups was insufficient for reliable sex-specific estimates. After the exclusion of 29 men, the final analytical sample comprised 160 women. Participants were recruited as a non-probability convenience sample of volunteers; consequently, selection bias and healthy-volunteer effects were possible and were considered when interpreting both between-group and duration-based findings.

Eligibility criteria for the older groups included the ability to ambulate independently without a walking aid, the ability to understand and follow the test instructions, and the provision of written informed consent. The registered study protocol specified a lower age threshold of ≥65 years for the older groups. During study implementation, the operational recruitment threshold was broadened to ≥60 years to better reflect the source population attending retirement-oriented community programs, including senior dance. Accordingly, women aged ≥60 years were eligible for the older study groups in the present analysis. This departure from the registered age criterion is reported transparently in Section 2.7 and considered in our discussion of the limitations.

Women aged 25–40 years were eligible for inclusion in the physically inactive younger reference group.

Participants were assigned to the senior dance group if they had attended organized senior dance classes for at least 3 months, with a minimum frequency of one session per week. All dance classes were led by instructors who had completed a standardized instructor training program based on the principles of the International Senior Dance Committee (ISDC).

Senior dance participants were recruited through certified senior dance instructors, physically inactive older adults through senior community clubs and faith-based community organizations, and physically inactive younger adults through the investigators’ personal networks.

Exclusion criteria included the regular use of a walking aid and any lower-limb injury or surgical procedure within the six months preceding data collection.

Although both women and men volunteered for the study, the analyses were restricted post hoc to women because the male subgroup sizes were too small to support reliable sex-specific estimates or meaningful assessment of sex-by-group interactions. This analytical restriction was used to avoid conclusions based on highly imprecise male subgroup estimates and limits direct generalization of the present findings to older men.

### 2.3. Study Size

No a priori sample-size calculation was performed. The study sample comprised all eligible participants recruited and assessed during the predefined data-collection period from September 2025 to March 2026. Following the post hoc restriction of the present analyses to women because male subgroup sizes were insufficient for reliable sex-specific estimates, the final analytical sample comprised 160 women. Accordingly, the analytical sample size was determined by the number of eligible women available during the study period rather than by a prospectively specified statistical power target.

### 2.4. Study Groups and Physical Activity Classifications

**Figure A1:**
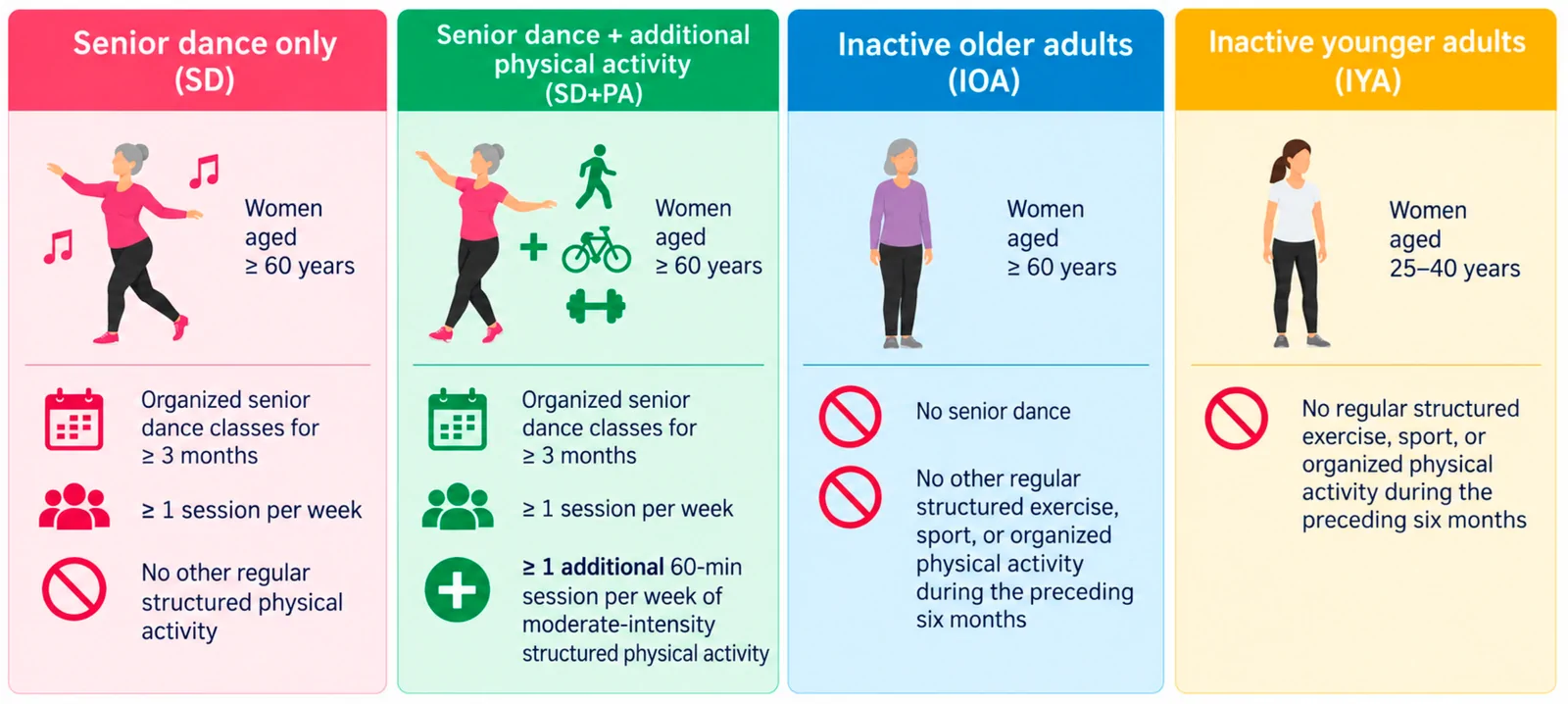
Graphical summary of the operational definitions and classification criteria of the four study groups. SD, senior dance only; SD+PA, senior dance plus additional regular physical activity; IOA, inactive older adults; IYA, inactive younger adults. The figure was prepared by the authors with AI-assisted graphical support using ChatGPT (OpenAI; GPT 5.6 Sol); all scientific content, eligibility criteria, labels, and final presentation were specified, reviewed, and verified by the authors.

Participants were classified into four groups according to age, senior dance participation, and engagement in other regular structured physical activity. The operational definitions of the study groups are described below and are also summarized graphically in Appendix A (Figure A1).

1. Senior dance only (SD): women aged ≥ 60 years who had participated in organized senior dance classes for at least three months, attended at least one session per week, and did not report any other regular structured physical activity.
2. Senior dance plus additional regular physical activity (SD+PA): women aged ≥ 60 years who met the senior dance participation criteria and additionally reported engaging in at least one 60-min session of moderate-intensity structured physical activity per week.
3. Inactive older adults (IOA): women aged ≥ 60 years who had not participated in senior dance or any other regular structured exercise, sport, or organized physical activity during the preceding six months.
4. Inactive younger adults (IYA): women aged 25–40 years who had not participated in regular structured exercise, sport, or organized physical activity during the preceding six months.

The additional-activity criterion for the SD+PA group was used solely for study-group classification and should not be interpreted as evidence of adherence to the World Health Organization recommendation of 150–300 min per week of moderate-intensity aerobic physical activity.

For classification purposes, structured physical activity included organized exercise classes, sport, planned fitness training, cycling or walking performed specifically for exercise, and other regularly undertaken planned moderate-intensity activities. Incidental walking, active transport, household tasks, and occupational activities were not used to classify participants as SD+PA unless they were reported as planned regular exercise meeting the predefined frequency, duration, and intensity criteria.

Accordingly, the terms “inactive older adults” and “inactive younger adults” refer to the absence of regular structured physical activity rather than complete physical inactivity. Participants in these groups may still have engaged in incidental walking, transport-related movement, household activities, or other activities of daily living; total lifestyle physical activity was not quantified comprehensively.

### 2.5. Measurement Protocol and Outcome Measures

All assessments were conducted by four licensed physiotherapists with 22, 12, 10, and 10 years of clinical experience in geriatric physiotherapy, respectively. The assessment followed a standardized measurement protocol and provided standardized verbal instructions and demonstrations before each performance test, as well as information that participants could withdraw from the measurement at any time without providing a reason. The tests were administered in a fixed order, with rest periods provided between assessments as needed to minimize fatigue.

Participants were assessed while wearing comfortable clothing and their usual stable footwear.

The outcome measures were categorized as mental well-being; functional performance (functional mobility, lower-limb functional performance, and functional aerobic endurance); muscle strength and body composition; and flexibility.

#### 2.5.1. Mental Well-Being

Mental well-being was assessed using the Hungarian four-response version of the five-item WHO Well-Being Index (WBI-5), validated in a representative Hungarian population by Susánszky et al. [12]. The WBI-5 was treated as a unidimensional index of positive subjective well-being rather than as a set of separate subscales. Its five items address positive mood, calmness/relaxation, vitality, feeling fresh and rested, and interest in daily life. Participants rated each positively worded statement on a four-response scale from 0 to 3. Item scores were summed to obtain a total score ranging from 0 to 15, with higher scores indicating more favorable mental well-being. This Hungarian four-response WBI-5 should be distinguished from the currently more widely used six-response WHO-5, which yields a raw score from 0 to 25. The original 0–15 total score was used in the present analyses and was not transformed to a percentage score.

The Hungarian WBI-5 demonstrated good internal consistency (Cronbach’s α = 0.85) in the Hungarostudy 2002 validation study [12].

#### 2.5.2. Functional Mobility

Functional mobility was assessed using the Timed Up and Go (TUG) test, originally developed as a measure of basic functional mobility in older adults [13].

Participants were instructed to rise from a standard chair with a seat height of 46 cm, walk 3 m at their usual safe pace, turn around a marker, return to the chair, and sit down. Timing began on the command “Go” and stopped when the participant’s back contacted the chair backrest. Participants completed one familiarization trial followed by two recorded trials. The value used in the analysis was the mean of the two trials. Participants wore their usual footwear. Walking aids were not permitted because regular use of a walking aid was an exclusion criterion. Shorter completion times indicate better functional mobility.

The TUG was interpreted as an integrated measure of functional mobility involving standing transfer, walking, turning, and controlled sitting, rather than as an isolated measure of balance or dynamic postural control.

The TUG test has demonstrated excellent test–retest reliability in community-dwelling older adults, with intraclass correlation coefficients (ICC) ranging from 0.95 to 0.99.

#### 2.5.3. Lower-Limb Functional Performance

Lower-limb functional performance was assessed using the 30-Second Chair Stand Test, an established performance-based measure developed for community-dwelling older adults [14,15].

Participants sat in the middle of a standard chair with their feet placed on the floor and their arms crossed over their chests. They were instructed to rise to a fully standing position and return to a fully seated position as many times as possible within 30 s without using their arms.

Participants completed one familiarization repetition before the recorded test. One recorded trial was performed. The total number of correctly completed stands was used in the analysis, with a higher number indicating better lower-limb functional performance.

The test was interpreted as a measure of repeated chair-rise performance involving lower-limb strength, power, muscular endurance, coordination, and transfer ability, rather than as a direct measure of maximal lower-limb strength.

The 30-Second Chair Stand Test has demonstrated excellent test–retest reliability in community-dwelling older adults (r = 0.89; women: r = 0.92) and excellent inter-rater reliability (r = 0.95) [14,15,16].

#### 2.5.4. Functional Aerobic Endurance

Functional aerobic endurance was assessed using the 2-Minute Step Test (2MST) from the Senior Fitness Test battery [14].

Participants marched in place for two minutes, raising each knee to a target height corresponding to the midpoint between the patella and the anterior superior iliac spine. Before the recorded test, they completed a brief familiarization trial of approximately one full stepping cycle to ensure correct execution of the movement. Participants were allowed to reduce their pace or briefly stop if necessary, while the timer continued to run.

The outcome used in the analyses was the total number of correctly completed right-knee elevations reaching the target height during the 2-min test period, with higher values indicating better functional aerobic endurance.

The 2MST has demonstrated excellent test–retest reliability in community-dwelling older adults (ICC = 0.90) [17].

#### 2.5.5. Handgrip Strength

Handgrip dynamometry has demonstrated excellent test–retest reliability in community-dwelling older adults, with intraclass correlation coefficients ranging from 0.91 to 0.99 [18].

Handgrip strength was measured using a CAMRY digital hand dynamometer (model E108H; Zhongshan CAMRY Electronic Co., Ltd., Zhongshan, Guangdong, China) with the handle adjusted to ensure the proximal interphalangeal joint of the index finger was flexed at a 90-degree angle.

Participants were seated with the shoulder adducted and neutrally rotated, the elbow flexed to 90°, the forearm in a neutral position, and the wrist positioned between 0–30° extension and 0–15° ulnar deviation. The feet were supported on the floor, and the tested arm was not supported against the trunk or chair.

The dominant hand was defined as the hand normally used for writing. Participants performed two maximal voluntary contractions with the dominant hand. Each contraction was sustained for 5 s, with 30 s of rest between trials. Standardized verbal encouragement was provided.

The handgrip value used in the analysis was the mean of the two trials. Results were recorded in kilograms. Higher values indicated greater handgrip strength [18].

#### 2.5.6. Body Composition

Body composition was assessed using an OMRON BF511 body composition monitor (HBF-511B-E; OMRON Healthcare Co., Ltd., Kyoto, Japan). According to the manufacturer’s technical documentation, the device applies a low-level single frequency (50 kHz) electrical current through eight hand and foot electrodes and combines bioelectrical impedance measurements with entered personal characteristics to generate device-specific estimates of body composition [19].

Participants were assessed barefoot and wearing light clothing. Measurements were performed in the early afternoon, between 13:00 and 17:00. Participants were measured at least 30 min after their most recent food or fluid intake, were instructed to abstain completely from alcohol for 24 h before the assessment and were asked to empty their bladder immediately before measurement. Recent strenuous exercise was assessed by self-report. Height, age, and sex were entered into the device according to the manufacturer’s instructions.

During measurement, participants stood with both feet positioned on the foot electrodes and held the hand electrodes with their arms extended horizontally until the assessment was completed. The outcome used in the analysis was the device-estimated skeletal muscle percentage. This value was not interpreted as a direct measurement of skeletal muscle mass and was treated as an algorithm-derived body-composition estimate rather than as a performance-based functional test.

The manufacturer’s reference classifications for skeletal muscle percentage apply to adults aged 18–80 years [19]. Values from participants older than 80 years were retained in the analyses because these participants met the study eligibility criteria, but the manufacturer’s age-specific reference classifications were not applied to them. No age-subgroup sensitivity analysis was prespecified or performed. Accordingly, device-estimated skeletal muscle percentage values in participants older than 80 years should be interpreted cautiously and should not be considered validation of the manufacturer’s reference categories beyond 80 years of age.

#### 2.5.7. Flexibility

Lower- and upper-body flexibility were assessed using the Chair Sit-and-Reach and Back Scratch tests, respectively.

##### Lower-Body Flexibility

Lower-body flexibility was assessed using the Chair Sit-and-Reach Test from the Senior Fitness Test battery [14].

Participants sat near the front edge of a standard chair with the preferred leg extended, the heel placed on the floor, the ankle dorsiflexed, and the knee maintained in extension. With the hands overlapping, participants reached slowly towards the toes of the extended leg.

The distance between the tips of the middle fingers and the tip of the toe was measured in centimeters. Negative values indicated that the fingertips did not reach the toes, zero indicated that the fingertips reached the toes, and positive values indicated reaching beyond the toes. Higher and more positive values indicated better flexibility.

The test was performed one time on the preferred side. The value used in the analysis was the result from the preferred side.

##### Upper-Body Flexibility

Upper-body flexibility was assessed using the Back Scratch Test from the Senior Fitness Test battery [14].

Participants placed one hand over the ipsilateral shoulder and reached down the back while moving the other hand behind the waist and upwards along the back, attempting to touch or overlap the middle fingers.

The distance between the tips of the middle fingers was measured in centimeters. Negative values indicated a gap between the fingers, zero indicated that the fingertips touched, and positive values indicated overlap. Higher and more positive values indicated better flexibility. The test was performed once, with the dominant arm overhead. This trial served as the basis for the analysis.

The Back Scratch Test has demonstrated excellent test–retest reliability in community-dwelling older adults (ICC = 0.95–0.99).

#### 2.5.8. Physical Activity and Senior Dance Participation

##### Physical Activity

Physical activity information was collected using selected questions adapted from the validated Hungarian short form of the International Physical Activity Questionnaire (IPAQ-SF) [20,21,22]. Because only the first four items were administered, the complete IPAQ-SF scoring protocol was not applied, and participants were not classified according to standard IPAQ activity categories or according to whether they met World Health Organization physical-activity recommendations. The selected questions were used to record the type, frequency, duration, and perceived intensity of physical activities performed outside senior dance.

Information on senior dance participation included total participation duration in months and the current number of senior dance sessions attended per week. Where available, the usual duration of each senior dance session was also recorded. Weekly attendance frequency was analyzed as a measure of current participation frequency and was not interpreted as weekly exercise dose because total minutes and intensity were not quantified consistently.

Incidental or lifestyle activities were described separately and were not treated as equivalent to regular structured exercise.

##### Senior Dance Participation

Data on senior dance participation included participation duration (months) and current weekly attendance frequency. Participants also reported the type, frequency, and duration of regular physical activities performed outside senior dance; these data were used for the predefined study-group classification.

The SD+PA designation indicated senior dance plus at least one additional 60-min session of moderate-intensity structured physical activity per week. This criterion was used only for study-group classification and should not be interpreted as evidence that participants met current WHO physical-activity recommendations [23].

### 2.6. Statistical Analyses

Overall differences in mental well-being and physical outcomes among the four study groups were examined using Welch’s one-way analysis of variance as a supplementary global test of between-group heterogeneity. Distributional shape was inspected using z values for skewness, and homogeneity of variances was assessed using Levene’s test. Statistical significance was defined as *p* < 0.05 before multiplicity adjustment where applicable.

All eight outcomes were analyzed on their observed continuous scales. No outcome was dichotomized or categorized for the inferential analyses because no clinically justified cut points had been prespecified and categorization would have discarded information. Consequently, odds ratios were not an appropriate effect measure for the principal study questions.

Because the study was guided by prespecified, theory-driven questions, group differences were examined using planned contrasts. Planned contrasts allow direct evaluation of specific hypotheses defined before interpretation of the results rather than testing all possible pairwise comparisons [24,25].

Welch-type inference was used because the groups were unequal in size and variance heterogeneity was observed for several outcomes. For each planned contrast, separate-variance standard errors and Welch–Satterthwaite approximate degrees of freedom were used.

The contrasts were described as prespecified planned contrasts, not as orthogonal contrasts.

For each planned contrast, the signed contrast estimate, Welch–Satterthwaite standard error, t statistic, approximate degrees of freedom, 95% confidence interval, unadjusted *p* value, and Benjamini–Hochberg-adjusted *p* value were reported.

Because each planned contrast addressed a distinct prespecified research question, C1, C2, and C3 were treated as separate inferential families. Within each contrast family, the eight outcome-specific *p* values were adjusted using the Benjamini–Hochberg (BH) false discovery rate procedure [26]. No additional correction was applied across the three contrast families. The duration analysis formed a separate inferential family: Model 2 was designated as the principal adjusted duration model, and BH correction was applied jointly across its eight outcome-specific tests. Models 0 and 1 were retained as supporting analyses showing the effect of sequential adjustment and were not subjected to multiplicity correction. Both unadjusted *p* values and BH-adjusted *p* values (q values) were reported, with q < 0.05 used as the FDR-adjusted significance criterion.

The first contrast (Contrast 1, C1) compared the mean of the physically inactive older group with the combined, equally weighted mean of the two senior dance groups and the physically inactive younger group using coefficients (+1, +1, +1, −3). This contrast was intended as a broad exploratory comparison between IOA and groups characterized by either younger age or regular senior dance participation.

Because the younger reference group and the senior dance groups differ for fundamentally different reasons, C1 intentionally mixes age and activity/dance status. It therefore cannot identify a dance-specific association.

Accordingly, C1 was interpreted descriptively and was not used to estimate the independent effect of senior dance, physical activity, or age.

The second contrast (Contrast 2, C2) compared the combined mean of the two senior dance groups with the physically inactive younger group using coefficients (+1, +1, −2, 0). This comparison provided exploratory age-related context and was not designed as an equivalence or non-inferiority test; therefore, a non-significant result was interpreted as insufficient evidence of a difference, not as evidence of equivalence [27].

The younger group therefore served as an age-based reference for functional performance rather than as a normative or equivalence benchmark.

The third contrast (Contrast 3, C3) compared older adults participating exclusively in senior dance with those who combined senior dance with additional regular physical activity using coefficients (+1, −1, 0, 0). This contrast examined whether the measured outcomes differed between the two senior dance groups under the present operational definition of additional regular physical activity.

Standardized effect sizes for the planned contrasts were expressed as Hedges’ g. IBM SPSS Statistics version 30.0 (IBM Corp., Armonk, NY, USA) calculated the effect sizes using the pooled within-group standard deviation across all four study groups with a small-sample correction. To obtain effect sizes corresponding to differences between weighted group means, the contrast coefficients were normalized such that the positive weights summed to +1 and the negative weights to −1. Accordingly, effect-size estimates obtained for C1 and C2 using the original SPSS coefficient scales were divided by 3 and 2, respectively; C3 required no rescaling. Absolute values of approximately 0.20, 0.50, and 0.80 were used only as descriptive benchmarks for small, moderate, and large standardized effects and were not treated as thresholds of clinical importance [28].

Subsequent analyses were restricted to the senior dance-only group (SD; *n* = 66). Associations between senior dance participation duration (months) and the eight outcomes were examined using three progressively adjusted rank-correlation models. Model 0 consisted of bivariate Spearman correlations. Model 1 estimated partial Spearman correlations adjusted for current weekly senior dance attendance frequency, and Model 2 estimated partial Spearman correlations adjusted for both weekly attendance frequency and chronological age.

Partial Spearman correlations were implemented by rank-transforming the exposure, outcomes, and covariates using mean ranks for tied observations and then calculating Pearson partial correlations between the ranked variables.

Model 2 was regarded as the principal adjusted duration analysis, whereas Models 0 and 1 were supporting analyses. For Model 2, 95% confidence intervals for the partial correlation coefficients were calculated using Fisher’s z transformation, accounting for the two control variables. Two-sided *p* values were reported. These models estimate monotonic associations and do not estimate causal effects, event probabilities, or odds ratios.

For descriptive interpretation of association magnitude, absolute correlation coefficients were classified as negligible (<0.10), weak (0.10–0.29), moderate (0.30–0.49), or strong (≥0.50) [29]. These thresholds were used as general descriptive guidelines rather than as indicators of clinical importance.

Statistical analyses were conducted using IBM SPSS Statistics version 30.0 (IBM Corp., Armonk, NY, USA).

### 2.7. Ethics

The study was conducted in accordance with the Declaration of Helsinki. Ethical approval was obtained from the Institutional Review Board before participant recruitment, and all participants provided written informed consent prior to enrolment. The study was registered at ClinicalTrials.gov (Identifier: NCT07170241).

The registered study protocol encompassed a broader set of outcomes, including gait-related measures, and specified an eligibility criterion of ≥65 years for the older groups. During study implementation, participants aged 60–64 years were also recruited under the operational ≥60-year criterion described in Section 2.2. This departure from the registered age criterion is reported transparently as a protocol deviation and is considered when interpreting comparability and generalizability.

## 3. Results

### 3.1. Participant Characteristics

**Figure 1:**
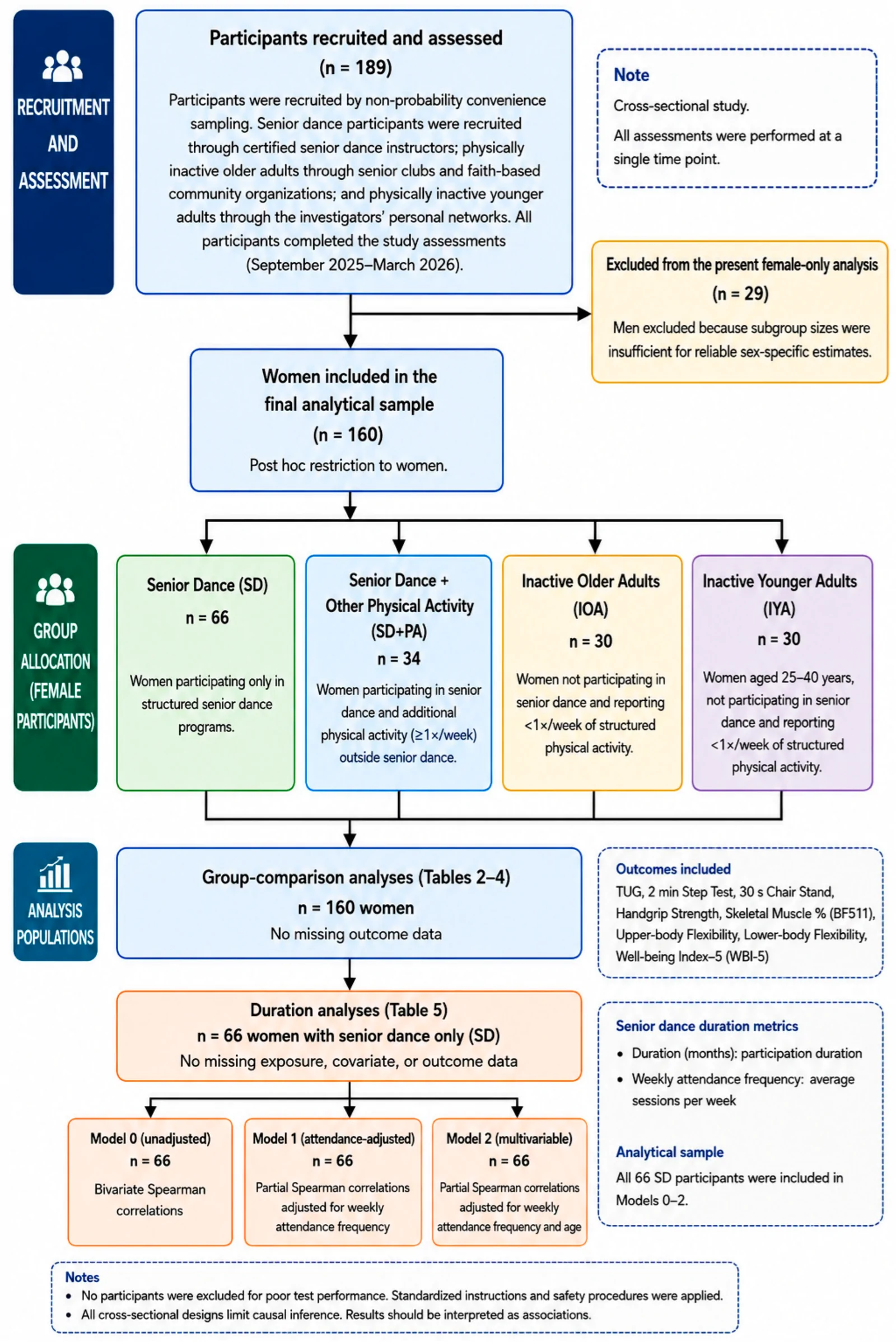
Flow of participants through recruitment, group allocation, and analysis. Group-comparison analyses are presented in Table 2, Table 3 and Table 4, and duration analyses are presented in Table 5. The figure was prepared by the authors with generative-AI-assisted graphical support using ChatGPT (GPT-5.6 Sol; OpenAI), with image-generation support provided by ChatGPT Images 2.0. All study information, participant numbers, labels, and analytical pathways were specified and verified by the authors.

A total of 189 participants were recruited using non-probability convenience sampling and completed the study assessments between September 2025 and March 2026. Senior dance participants were recruited through certified senior dance instructors, physically inactive older adults were recruited through senior clubs and faith-based community organizations, and physically inactive younger adults were recruited through the investigators’ personal networks. Because male subgroup sizes were insufficient for reliable sex-specific estimates, the present analysis was restricted post hoc to women. After exclusion of 29 men, the final analytical sample comprised 160 women: 66 in the senior dance-only group (SD), 34 in the senior dance plus additional regular physical activity group (SD+PA), 30 physically inactive older women (IOA), and 30 physically inactive younger women (IYA). (Table 1)There were no missing outcome data in the final analytical sample; all 160 women contributed complete data to the descriptive and group comparison analyses (Table 2, Table 3 and Table 4). Among the 66 women in the SD group, there were no missing data for participation duration, current weekly attendance frequency, age, or any of the eight outcomes; consequently, all 66 participants were included in Models 0–2 of the duration analyses (Table 5). Participant flow is shown in Figure 1.

The sociodemographic and health-related characteristics of the participants are summarized in Table 1. Previous falls were reported more frequently in IOA (20/30, 66.7%) than in SD (8/66, 12.1%) or SD+PA (5/34, 14.7%), indicating substantial baseline differences and possible greater functional vulnerability in the inactive older group.

### 3.2. Descriptive Analysis

Descriptive statistics for mental well-being and physical outcomes across the four study groups are presented in Table 2. Within the older groups, the unadjusted mean values of both senior dance groups were more favorable than those of IOA for all eight outcomes. These comparisons are descriptive only and should not be interpreted as dance-specific effects because the groups were not randomized and differed in baseline characteristics, including previous fall history and incompletely characterized total physical activity. The device-estimated skeletal muscle percentage should also be interpreted separately from the directly observed performance-based tests because it is an algorithm-derived bioimpedance estimate.

**Table 2:** Descriptive statistics for mental well-being and physical outcomes across the four study groups.

Outcomes	SD (*n* = 66)	SD+PA (*n* = 34)	IOA (*n* = 30)	IYA (*n* = 30)
**Mental well-being**				
WBI-5 scores	11.20 ± 2.33	11.21 ± 2.07	7.03 ± 2.39	9.73 ± 2.42
**Functional performance**				
Timed Up and Go (s)	7.43 ± 1.37	6.94 ± 1.12	13.66 ± 4.93	6.37 ± 0.68
30-s Chair Stand (repetitions)	13.67 ± 3.46	15.18 ± 3.63	9.53 ± 2.79	15.63 ± 2.68
2-min Step Test (steps)	86.61 ± 21.12	94.09 ± 18.69	57.57 ± 18.84	96.93 ± 13.48
**Muscle strength and body composition**				
Handgrip strength (kg)	24.10 ± 5.29	24.09 ± 4.77	17.29 ± 5.81	32.34 ± 8.12
Device-estimated skeletal muscle percentage (%)	27.60 ± 3.28	27.63 ± 3.19	25.34 ± 3.60	30.23 ± 6.18
**Flexibility**				
Lower-body flexibility (cm)	−11.53 ± 10.65	−8.54 ± 9.46	−16.87 ± 11.07	−18.57 ± 11.46
Upper-body flexibility (cm)	−7.15 ± 10.34	−5.69 ± 9.76	−10.48 ± 10.24	−0.27 ± 9.67

Note: Boldface and shading denote outcome-domain headings. Data are presented as mean ± standard deviation. SD, senior dance only; SD+PA, senior dance plus additional regular physical activity; IOA, physically inactive older adults; IYA, physically inactive younger adults; WBI-5, five-item WHO Well-Being Index; TUG, Timed Up and Go; 2MST, 2-Minute Step Test. Lower TUG values indicate better functional mobility; higher values indicate more favorable performance for the remaining outcomes.

### 3.3. Overall Between-Group Differences

Welch’s one-way analysis of variance indicated statistically significant overall differences among the four study groups for all eight outcomes examined (Table 3; all *p* ≤ 0.002). Significant between-group variation was observed for mental well-being, functional mobility, lower-limb functional performance, functional aerobic endurance, handgrip strength, device-estimated skeletal muscle percentage, and lower- and upper-body flexibility.

Specifically, significant overall group differences were observed for WBI-5 score, Welch’s F(3, 71.829) = 24.245, *p* < 0.001; Timed Up and Go performance, F(3, 72.197) = 27.528, *p* < 0.001; 30-Second Chair Stand performance, F(3, 74.668) = 28.484, *p* < 0.001; 2-Minute Step Test performance, F(3, 75.599) = 30.895, *p* < 0.001; handgrip strength, F(3, 69.081) = 23.239, *p* < 0.001; device-estimated skeletal muscle percentage, F(3, 67.885) = 5.378, *p* = 0.002; lower-body flexibility, F(3, 71.518) = 6.368, *p* < 0.001; and upper-body flexibility, F(3, 72.545) = 5.614, *p* = 0.002.

The omnibus analyses established overall between-group heterogeneity but did not identify which prespecified group comparisons accounted for the differences. The hypothesis-directed group comparisons were therefore evaluated using the planned contrasts described in Section 3.4.

**Table 3:** Welch’s one-way analyses of variance for mental well-being and physical outcomes across the four study groups.

	Homogeneity of Variance		Welch ANOVA			
Outcome	Levene F	Levene *p*	Welch F	df1	df2	*p*
**Mental well-being**						
WBI-5 score	0.141	0.935	24.245	3	71.829	<0.001
**Functional performance**						
Timed Up and Go	39.568	<0.001	27.528	3	72.197	<0.001
30-s Chair Stand	1.340	0.264	28.484	3	74.668	<0.001
2-min Step Test	2.060	0.108	30.895	3	75.599	<0.001
**Muscle strength and body composition**						
Handgrip strength	5.826	<0.001	23.239	3	69.081	<0.001
Device-estimated skeletal muscle percentage	5.312	0.002	5.378	3	67.885	0.002
**Flexibility**						
Lower-body flexibility	0.120	0.948	6.368	3	71.518	<0.001
Upper-body flexibility	0.222	0.881	5.614	3	72.545	0.002

Note: Boldface and shading denote outcome-domain headings. Levene’s test was based on deviations from the group means. Welch’s one-way analysis of variance was used as a supplementary global test of between-group differences because group sizes were unequal and homogeneity of variance was violated for several outcomes. The Welch omnibus tests were not used as gatekeeping tests for the prespecified planned contrasts. Conventional η^2^ was not reported because it is based on the equal-variance ANOVA decomposition and is not directly compatible with the Welch F statistic.

### 3.4. Prespecified Planned Contrasts

In Contrast 1, the combined, equally weighted mean of SD, SD+PA, and IYA was more favorable than the IOA mean for seven of the eight outcomes after FDR correction. The combined comparator showed higher WBI-5 scores, shorter TUG times, more Chair Stand repetitions, higher 2-Minute Step Test performance, greater handgrip strength, higher device-estimated skeletal muscle percentage, and better upper-body flexibility. Lower-body flexibility was not significantly different (*p* = 0.083; q = 0.083). Because C1 intentionally combines younger age and senior dance participation, it was interpreted as exploratory and not as evidence of a senior dance-specific association (Table 4).

**Table 4:** Prespecified Welch–Satterthwaite planned contrasts across mental well-being and physical outcomes.

Outcome	C1: Mean of SD, SD+PA, and IYA Minus IOA				C2: Mean of SD and SD+PA Minus IYA				C3: SD Minus SD+PA			
	Estimate [95% CI]	t (df)	Hedges g	*p*/q	Estimate [95% CI]	t (df)	Hedges g	*p*/q	Estimate [95% CI]	t (df)	Hedges g	*p*/q
**Mental well-being**												
WBI-5 (scores)	3.68 [2.70, 4.65]	7.597 (43.514)	1.59	<0.001/<0.001	1.47 [0.47, 2.47]	2.92 (45.21)	0.63	0.005/0.008	−0.01 [−0.92, 0.90]	−0.020 (74.032)	−0.00	0.984/0.992
**Functional performance**												
Timed Up and Go (s)	−6.75 [−8.60, −4.90]	−7.456 (29.648)	−2.82	<0.001/<0.001	0.81 [0.46, 1.17]	4.555 (87.021)	0.34	<0.001/<0.001	0.49 [−0.02, 1.00]	1.917 (79.218)	0.21	0.059/0.197
30-s Chair Stand (repetitions)	5.29 [4.11, 6.48]	8.958 (50.698)	1.62	<0.001/<0.001	−1.21 [−2.45, 0.02]	−1.960 (63.415)	−0.37	0.054/0.054	−1.51 [−3.02, −0.003]	−2.002 (63.941)	−0.46	0.050/0.197
2-min Step Test (steps)	34.98 [27.32, 42.63]	9.217 (42.377)	1.84	<0.001/<0.001	−6.59 [−12.99, −0.18]	−2.051 (70.515)	−0.35	0.044/0.050	−7.48 [−15.70, 0.74]	−1.813 (74.386)	−0.39	0.074/0.197
**Muscle strength and body composition**												
Handgrip strength (kg)	9.56 [7.10, 12.01]	7.824 (48.403)	1.61	<0.001/<0.001	−8.24 [−11.43, −5.05]	−5.241 (36.442)	−1.39	<0.001/<0.001	0.01 [−2.07, 2.09]	0.010 (73.122)	0.00	0.992/0.992
Device-estimated skeletal muscle percentage (%)	3.14 [1.56, 4.73]	3.973 (54.441)	0.78	<0.001/<0.001	−2.62 [−5.01, −0.22]	−2.219 (34.384)	−0.65	0.033/0.044	−0.03 [−1.39, 1.32]	−0.051 (68.427)	−0.01	0.959/0.992
**Flexibility**												
Lower-body flexibility (cm)	3.99 [−0.55, 8.52]	1.773 (43.567)	0.37	0.083/0.083	8.53 [3.82, 13.24]	3.647 (44.129)	0.80	<0.001/0.002	−2.99 [−7.14, 1.17]	−1.431 (74.147)	−0.28	0.157/0.313
Upper-body flexibility (cm)	6.11 [1.91, 10.31]	2.937 (43.790)	0.60	0.005/0.006	−6.16 [−10.28, −2.03]	−2.995 (50.598)	−0.61	0.004/0.008	−1.46 [−5.67, 2.74]	−0.695 (70.259)	−0.14	0.489/0.783

Note: Boldface and shading denote outcome-domain headings. Contrast estimates are reported as normalized weighted-mean differences. C1 compared the mean of the SD, SD+PA, and IYA groups with the IOA group; C2 compared the mean of the SD and SD+PA groups with the IYA group; and C3 compared SD with SD+PA. Contrast tests were based on the SPSS separate-variance (“does not assume equal variances”) estimates, with Welch–Satterthwaite standard errors and approximate degrees of freedom. For C1, estimates and confidence limits obtained using coefficients (+1, +1, +1, −3) were divided by 3; for C2, those obtained using (+1, +1, −2, 0) were divided by 2; C3 (+1, −1, 0, 0) required no rescaling. This normalization does not alter the t statistics, degrees of freedom, or *p* values. Benjamini–Hochberg correction was applied separately across the eight outcomes within each contrast family, and the resulting FDR-adjusted *p* values are reported as q values. Standardized effect sizes are reported as Hedges’ g. Because the SPSS standardizer uses the pooled within-group standard deviation across all four groups, these values represent standardized linear-contrast effect sizes rather than conventional pairwise Hedges’ g values. For TUG, lower values indicate more favorable performance.

In Contrast 2, the combined senior dance groups had higher WBI-5 scores (*p* = 0.005; q = 0.008) and better lower-body flexibility (*p* < 0.001; q = 0.002) than the IYA group. Conversely, IYA showed more favorable TUG time (q < 0.001), greater handgrip strength (q < 0.001), higher skeletal muscle percentage (*p* = 0.033; q = 0.044), and better upper-body flexibility (*p* = 0.004; q = 0.008). Chair Stand performance did not differ significantly (*p* = 0.054; q = 0.054); the 2-Minute Step Test difference was nominally significant before correction (*p* = 0.044) but did not meet the FDR criterion after correction (q = 0.0503). This contrast tested for differences and did not establish equivalence between the older senior dance participants and the younger reference group (Table 4).

In Contrast 3, no SD versus SD+PA difference remained statistically significant after FDR correction. The Chair Stand contrast had an unadjusted *p* = 0.0495 but was not significant after BH adjustment (q = 0.197). The 2-Minute Step Test difference was not significant before or after correction (*p* = 0.074; q = 0.197). No FDR-significant differences were observed for WBI-5, TUG, handgrip strength, device-estimated skeletal muscle percentage, or either flexibility outcome. Thus, under the present operational definition and achieved subgroup size, no statistically significant additional association was detected for the heterogeneous supplementary regular physical activities reported by SD+PA participants (Table 4).

### 3.5. Associations with Duration of Senior Dance Participation

Associations between the duration of senior dance participation and the study outcomes were examined among the 66 women in the SD group using bivariate and progressively adjusted rank-correlation models (Table 5).

Among the 66 women in the SD group, there were no missing data for participation duration, weekly attendance frequency, age, or any of the eight outcomes; all 66 participants were therefore included in Models 0–2.

In Model 0, participation duration correlated with higher WBI-5 scores (r_s_ = 0.309, *p* = 0.011), shorter TUG times (r_s_ = −0.372, *p* = 0.002), more Chair Stand repetitions (r_s_ = 0.401, *p* < 0.001), and higher 2-Minute Step Test performance (r_s_ = 0.415, *p* < 0.001). No statistically significant bivariate correlations were observed for handgrip strength, device-estimated skeletal muscle percentage, or lower- or upper-body flexibility.

Adjustment for current weekly senior dance attendance frequency in Model 1 produced only modest changes in the correlations. Participation duration remained correlated with WBI-5 score (partial r_s_ = 0.295, *p* = 0.017), TUG performance (partial r_s_ = −0.357, *p* = 0.003), Chair Stand performance (partial r_s_ = 0.396, *p* = 0.001), and 2-Minute Step Test performance (partial r_s_ = 0.385, *p* = 0.002).

In the principal Model 2 analysis, after adjustment for current weekly senior dance attendance frequency and chronological age, participation duration correlated with more favorable functional mobility as assessed by TUG (partial r_s_ = −0.428, 95% CI −0.610 to −0.204, *p* < 0.001, q = 0.0013), higher repeated chair-rise performance (partial r_s_ = 0.425, 95% CI 0.200 to 0.607, *p* < 0.001, q = 0.0013), and higher functional aerobic endurance on the 2-Minute Step Test (partial r_s_ = 0.423, 95% CI 0.198 to 0.606, *p* < 0.001, q = 0.0013). The WBI-5 correlation was positive before multiplicity adjustment (partial r_s_ = 0.271, 95% CI 0.027 to 0.485, *p* = 0.030) but did not remain significant after BH correction (q = 0.061). Correlations with handgrip strength, device-estimated skeletal muscle percentage, lower-body flexibility, and upper-body flexibility were not statistically significant after correction (all q ≥ 0.139). These findings describe adjusted cross-sectional correlations and do not establish a temporal or causal effect of participation duration (Table 5).

**Table 5:** Associations between duration of senior dance participation and mental well-being and physical outcomes in the senior dance-only group.

	Model 0		Model 1		Model 2			
Outcome	r_s_	*p*	Partial r_s_	*p*	Partial r_s_	95% CI	*p*	BH q
**Mental well-being**								
WBI-5 scores	0.309	0.011	0.295	0.017	0.271	0.027–0.485	0.030	0.0605
**Functional performance**								
Timed Up and Go (s)	−0.372	0.002	−0.357	0.003	−0.428	−0.610–−0.204	<0.001	0.0013
30-s Chair Stand (repetitions)	0.401	<0.001	0.396	0.001	0.425	0.200–0.607	<0.001	0.0013
2-min Step Test (steps)	0.415	<0.001	0.385	0.002	0.423	0.198–0.606	<0.001	0.0013
**Muscle strength and body composition**								
Handgrip strength (kg)	0.064	0.607	0.074	0.558	0.136	−0.114–0.369	0.285	0.3615
Device-estimated skeletal muscle percentage (%)	0.161	0.195	0.142	0.259	0.051	−0.197–0.293	0.687	0.6872
**Flexibility**								
Lower-body flexibility (cm)	0.149	0.233	0.190	0.130	0.216	−0.031–0.439	0.087	0.1391
Upper-body flexibility (cm)	0.132	0.289	0.115	0.361	0.127	−0.123–0.362	0.316	0.3615

Note: Model 0 represents bivariate Spearman rank correlations. Model 1 represents partial Spearman correlations adjusted for current weekly senior dance attendance frequency. Model 2 represents partial Spearman correlations adjusted for both weekly senior dance attendance frequency and chronological age. Partial Spearman correlations were implemented by rank-transforming the exposure, outcomes, and covariates using mean ranks for tied observations and calculating Pearson partial correlations between the ranked variables. All analyses included the 66 participants in the senior dance-only group. Two-sided *p*-values are reported. Model 0 and Model 1 were considered supporting analyses and were not adjusted for multiple testing. For Model 2, 95% confidence intervals for the partial rank correlations were calculated using Fisher’s *z* transformation, accounting for the two control variables (weekly senior dance attendance frequency and chronological age). For the principal Model 2 analysis, the eight outcome-specific *p*-values were adjusted jointly using the Benjamini–Hochberg procedure to control the false discovery rate at 5%; the resulting adjusted *p*-values are reported as q-values. For TUG, a negative correlation indicates that longer participation was associated with better functional mobility because shorter completion times indicate better performance. For all other outcomes, positive correlations indicate that longer participation was associated with higher outcome values. Boldface and shading denote outcome-domain headings. r_s_, Spearman rank correlation coefficient; BH, Benjamini–Hochberg; TUG, Timed Up and Go.

## 4. Discussion

### 4.1. Principal Findings

This cross-sectional study examined mental well-being and multiple dimensions of physical function among older women participating in senior dance, older women combining senior dance with additional regular physical activity, physically inactive older women, and a physically inactive younger reference group. The analyses provided two complementary perspectives: prespecified group contrasts and, within the senior dance-only group, correlations between participation duration and the measured outcomes. The principal interpretation is observational: the study characterizes outcome patterns among women who had already self-selected into these activity groups and does not estimate the causal effects of senior dance.

The group comparisons demonstrated marked heterogeneity across all eight outcomes. The first planned contrast showed that physically inactive older women differed from the combined mean of the two senior dance groups and the younger reference group across seven outcomes after FDR correction. However, because this contrast combined younger age and senior dance participation, the differences cannot be attributed specifically to dance or physical activity. More generally, physical inactivity in later life has been consistently associated with functional limitations, disability, and other adverse health outcomes [3,4].

Comparison of the combined senior dance groups with the younger reference group showed a mixed pattern. Older senior dance participants had higher WBI-5 scores and better lower-body flexibility, whereas younger women had better functional mobility, handgrip strength, device-estimated skeletal muscle percentage, and upper-body flexibility. Chair Stand performance did not differ significantly, and the nominal difference in the 2-Minute Step Test did not remain significant after FDR correction. Age-related variation across several components of functional fitness is well established [2], but the absence of a statistically significant difference does not demonstrate equivalence [27].

No outcome differed significantly between SD and SD+PA after correction for multiple testing. Previous comparisons between structured dance and other forms of physical activity likewise show substantial heterogeneity across modalities and outcomes [8,30]. Under the present broad operational definition and achieved subgroup size, no statistically significant additional association was detected for the heterogeneous supplementary activities reported by SD+PA participants. This finding does not establish that additional physical activity provides no benefit and should not be interpreted as evidence of equivalence.

The duration analyses showed a more specific pattern. After adjustment for current weekly senior dance attendance frequency and age, and after correction for multiple testing, participation duration correlated with more favorable TUG, 30-Second Chair Stand, and 2-Minute Step Test values. The WBI-5 correlation did not remain significant after FDR correction, and no FDR-significant adjusted correlations were identified for handgrip strength, device-estimated skeletal muscle percentage, or flexibility.

This concentration of robust associations within performance-based outcomes is broadly compatible with systematic reviews and meta-analyses of dance and physical function in older adults [5,6,31].

### 4.2. Duration of Participation and Functional Performance

The most consistent duration-related findings involved three measures of integrated functional performance. Participation duration showed moderate correlations with shorter TUG time, more Chair Stand repetitions, and higher 2-Minute Step Test performance after adjustment for weekly attendance frequency and age. These correlations remained statistically significant after FDR correction, but their cross-sectional nature precludes interpretation as improvement caused by longer participation.

The three tests capture different but overlapping dimensions of everyday physical function. TUG reflects functional mobility involving standing, walking, turning, and controlled sitting. The Chair Stand Test reflects repeated lower-limb functional performance, requiring force production, muscular endurance, coordination, and repeated transfers. The 2-Minute Step Test reflects functional aerobic endurance. Senior dance involves repeated stepping, weight transfer, directional changes, rhythmic coordination, and sustained movement. In a randomized trial specifically investigating Senior Dance, improvements were reported in balance, timed sit-to-stand performance, and short-distance walking [9]. Salsa-based training has likewise produced improvements in selected dynamic postural-control measures [32]. However, adaptations are not uniform: Kattenstroth et al. observed improvements in postural, sensorimotor, cognitive, and subjective well-being measures without corresponding improvements in cardiorespiratory performance [33].

Adjustment for weekly senior dance attendance frequency produced only modest changes in the observed correlations. Nevertheless, participation duration should not be interpreted as cumulative exercise dose because months of participation do not quantify total attendance, session intensity, interruptions, or accumulated dance exposure. Waugh et al. highlighted substantial variation in reporting dance dose and adherence [8], while McCullough and Han illustrate the methodological importance of distinguishing duration, frequency, and intensity when characterizing long-term dance exposure [34].

Adjustment for age modestly strengthened the correlations with TUG, Chair Stand, and 2-Minute Step Test performance. This indicates that age influenced the observed relationships but does not establish a causal mechanism. The cross-sectional design cannot distinguish whether sustained participation contributed to more favorable function or whether women with better functional capacity were more likely to initiate or maintain senior dance participation for longer periods. Statistical adjustment cannot establish temporal sequence or eliminate unmeasured confounding [11].

Healthy-participant and selective-retention effects are therefore particularly relevant. Older adults who volunteer for activity studies may differ systematically from nonparticipants in physical activity, functional status, and health characteristics [35,36].

Participants developing substantial mobility limitations, chronic illness, pain, or fatigue may have been more likely to discontinue senior dance, leaving healthier participants disproportionately represented among those with longer participation histories.

Favorable performance on functional tests should also not be interpreted as evidence that senior dance reduces actual fall incidence. The DAnCE cluster-randomized trial did not demonstrate a convincing reduction in falls [37], and a recent systematic review concluded that evidence for dance as an alternative to established fall-prevention interventions remains of very low certainty [38].

### 4.3. Mental Well-Being

Mental well-being showed a different pattern from functional performance. Senior dance is a recreational and social activity involving music, interpersonal interaction, motor learning, and shared participation. Qualitative studies involving older women participating in dance describe pleasure, social involvement, interpersonal connection, and perceived well-being as important aspects of participation [39,40].

At the group level, the senior dance groups showed relatively favorable WBI-5 scores, including higher scores than the younger inactive reference group in Contrast 2. This finding is compatible with the social, recreational, musical, and cognitively engaging characteristics of senior dance.

Within the SD group, participation duration showed a positive WBI-5 correlation before multiplicity correction, but this result did not remain statistically significant after BH adjustment (partial r_s_ = 0.271, *p* = 0.030, q = 0.061). This restrained interpretation is consistent with systematic reviews reporting potentially favorable psychological effects of dance but substantial heterogeneity across populations, comparators, and outcomes [41,42].

The duration analysis therefore did not provide FDR-controlled evidence of a correlation between participation duration and mental well-being.

This distinction is important. The favorable group-level WBI-5 profile may reflect several characteristics of senior dance participation—including social engagement, music, enjoyment, group belonging, and regular activity—but the present data cannot determine which factors contribute to the observed differences. Individuals with better psychological well-being or greater social engagement may also be more likely to initiate and maintain participation.

The present study did not measure social interaction, enjoyment, group belonging, or other psychosocial pathways as potential mediators. Consequently, these mechanisms remain plausible interpretations rather than demonstrated explanations [8,39,40].

### 4.4. Muscle Strength and Body Composition

Neither handgrip strength nor device-estimated skeletal muscle percentage showed a statistically significant correlation with senior dance participation duration in any of the three correlation models. These outcomes also showed no FDR-significant difference between SD and SD+PA. This domain-specific pattern is compatible with evidence that dance-related changes in integrated functional performance do not necessarily coincide with changes in isolated muscle-strength measures [6,32].

This pattern is physiologically plausible. Senior dance does not provide systematic progressive external resistance, whereas progressive resistance training is a well-established specific stimulus for increasing or preserving muscle strength and muscle mass in older adults [43]. Similarly, handgrip strength does not directly reflect the primary loading pattern of dance. The activity may therefore be more closely related to integrated movement performance than to maximal or near-maximal isolated muscle strength.

The OMRON BF511 outcome requires a different evidentiary interpretation from the performance tests. Skeletal muscle percentage is an algorithm-derived bioimpedance estimate rather than a directly observed functional outcome or a direct measurement of skeletal muscle mass. BIA estimates depend on hydration assumptions, measurement standardization, device characteristics, and population-specific algorithms [44,45]. The standardized measurement procedures used in this study reduce some sources of measurement variation but do not remove the device’s validity limitations, particularly beyond the manufacturer’s 18–80-year reference range. The absence of a duration correlation should therefore be interpreted only as the absence of a detectable relationship with this device-estimated parameter in the present sample.

### 4.5. Flexibility

Neither lower- nor upper-body flexibility showed a statistically significant adjusted correlation with participation duration. Lower-body flexibility had a weak positive Model 2 coefficient, but the evidence remained statistically inconclusive after FDR correction.

Targeted flexibility interventions can increase range of motion in older adults, although relationships with broader functional outcomes remain heterogeneous [46].

Dance interventions also differ substantially in their movement repertoire and range-of-motion demands [7].

The group contrasts also indicated that flexibility did not follow a uniform pattern. The combined senior dance groups showed better lower-body flexibility than the younger reference group, whereas upper-body flexibility favored the younger group. These findings suggest that flexibility should not be treated as a single generalized adaptation to senior dance and may depend on the specific movement demands of the activity, baseline characteristics, and other forms of exercise. The divergent lower- and upper-body flexibility findings argue against treating flexibility as a single generalized adaptation to senior dance [8,46].

### 4.6. Additional Regular Physical Activity

No SD versus SD+PA comparison remained statistically significant after FDR correction. The additional activities reported by SD+PA participants were heterogeneous in type, frequency, intensity, consistency, and likely physiological stimulus. Reviews comparing dance with other forms of physical activity likewise demonstrate substantial variation according to comparator and program design [8,30].

This result does not establish that additional physical activity provides no benefit. The SD+PA classification captured a minimum threshold of additional structured activity rather than total weekly activity volume, and the SD+PA subgroup was relatively small (*n* = 34). Exposure heterogeneity and imprecision may therefore have reduced the ability to detect between-group differences.

Although the point estimates for TUG, Chair Stand, and 2-Minute Step Test generally favored SD+PA, the evidence was insufficient to establish an additional association in the present sample.

The absence of statistically significant SD versus SD+PA differences cannot be interpreted as equivalence or non-inferiority, which require prespecified equivalence margins and dedicated testing procedures [27].

### 4.7. Interpretation of the Younger Reference Comparison

The younger inactive group provided age-related context rather than a normative or equivalence benchmark. Younger women performed better in TUG, handgrip strength, skeletal muscle percentage, and upper-body flexibility, whereas the senior dance groups showed higher WBI-5 scores and better lower-body flexibility. Chair Stand performance was not significantly different, and the Step Test result did not remain below the FDR threshold.

Age-specific reference data demonstrate substantial age-related variation across functional-fitness domains [2], while age-related reductions in muscle strength and mass are well documented [43].

Accordingly, statements that senior dance participants achieved “youthful” or equivalent functional capacity should be avoided. A non-significant conventional difference does not establish equivalence [27].

The contrast was not designed as an equivalence or non-inferiority analysis, and the younger group was itself an inactive comparison sample rather than a normative reference population.

### 4.8. Strengths and Limitations

The present study extends the literature by examining women engaged in established community-based senior dance rather than only a time-limited intervention. It also separates participation duration from current weekly attendance frequency and assesses a multidomain outcome profile spanning mental well-being, functional performance, handgrip strength, device-estimated body composition, and flexibility.

This study used a multidomain assessment framework encompassing mental well-being, functional mobility, lower-limb functional performance, functional aerobic endurance, handgrip strength, device-estimated skeletal muscle percentage, and flexibility.

The multidomain assessment showed that the most consistent duration correlations were concentrated in integrated functional-performance outcomes rather than distributed uniformly across mental well-being, muscle strength, body composition, and flexibility. This pattern should be viewed as hypothesis-generating rather than as evidence of efficacy.

The comparison of senior dance alone with senior dance plus additional regular physical activity also provided exploratory information on whether the broad operational SD+PA classification was associated with a detectable difference in the measured outcomes. Because the additional activities were heterogeneous and total activity volume was not quantified, this comparison should not be interpreted as a test of the benefit of additional exercise.

The analytical strategy combined prespecified group comparisons with duration-based analyses, used Welch-type inference where appropriate, reported estimates with confidence intervals, and addressed multiple testing using the Benjamini–Hochberg procedure within prespecified inferential families. Reporting was guided by STROBE principles for observational studies [11].

Several limitations remain. The cross-sectional design prevents causal inference and is particularly vulnerable to self-selection, healthy-participant, selective-retention, and baseline-confounding effects. Recruitment studies in older populations show that volunteers and nonparticipants may differ systematically in activity and health characteristics [35,36]. In the present sample, previous falls were substantially more frequent in IOA than in the two senior dance groups, suggesting that baseline functional vulnerability may have contributed to the observed between-group differences independently of senior dance participation. Total lifestyle physical activity and a comprehensive set of baseline confounders were not measured. Accordingly, the group contrasts should be interpreted as unadjusted observational comparisons; limited post hoc adjustment for one or two measured characteristics would not eliminate residual confounding and could overstate the degree of control achieved.

The primary analysis was restricted post hoc to women because male subgroup sizes were insufficient for reliable sex-specific estimates. Consequently, the findings cannot be generalized directly to older men, and potential sex-related differences or sex-by-group interactions could not be evaluated.

A further limitation concerns the age criterion used to define the older groups. Although the registered protocol specified a lower age threshold of ≥65 years, the operational recruitment criterion was broadened to ≥60 years during study implementation to better reflect the source population attending retirement-oriented community programs, including senior dance. Consequently, the analyzed older groups included women aged 60–64 years. This protocol deviation may limit direct comparability with studies or normative data applying a ≥65-year definition of older adulthood and should be considered when interpreting generalizability.

No a priori sample-size calculation was conducted, and the analytical sample was determined by the number of eligible participants available during the predefined recruitment period. Consequently, statistical precision was constrained by the achieved subgroup sizes, particularly for the smaller SD+PA group, and modest between-group differences may have been estimated imprecisely or remained undetected.

Physical activity and senior dance participation were predominantly self-reported. Current weekly attendance frequency should not be interpreted as exercise dose, and participation duration should not be interpreted as cumulative exposure [8,34], because total attendance, interruptions, session intensity, and accumulated activity volume were not quantified. Similarly, the SD+PA classification did not characterize the total physical activity profile sufficiently to compare overall activity dose across groups.

The duration analyses used the same complete sample of 66 women across Models 0–2, reducing the possibility that changes in coefficients across models resulted from changes in sample composition. Model 2 was designated as the principal adjusted analysis, and FDR correction was applied across its eight outcome tests. Nevertheless, the planned contrasts and partial rank-correlation models quantify between-group differences and associations; they do not estimate causal impact or odds of clinically defined outcomes. This distinction is especially important because the outcomes were retained on their continuous measurement scales rather than categorized post hoc.

### 4.9. Implications and Future Research

Contemporary systematic reviews support further investigation of dance as a multimodal physical activity for older adults while emphasizing heterogeneity across interventions and limitations in the certainty of the available evidence [5,6].

Consistent with the broader evidence base, the contribution of the present study is not to demonstrate efficacy or to establish the general proposition that longer physical activity is beneficial. Rather, it provides real-world observational evidence from established community-based senior dance, distinguishes participation duration from current weekly attendance frequency, compares senior dance alone with a broadly defined additional-activity subgroup, and identifies which measured domains were most consistently correlated with participation duration. In this sample, those correlations were concentrated in functional mobility, repeated chair-rise performance, and functional aerobic endurance, whereas evidence for mental well-being, handgrip strength, body composition, and flexibility was less robust. These cross-sectional correlations cannot establish that participation duration produced the observed differences.

Prospective longitudinal studies and adequately powered randomized controlled trials are therefore needed to determine whether senior dance contributes to changes in these outcomes over time. Future studies should characterize exposure more comprehensively, including session frequency, duration, intensity, attendance, interruptions, and physical activity performed outside the dance program; objective activity monitoring could further improve estimation of total physical activity exposure [8,34].

Larger, preferably multicenter samples should include sufficient numbers of women and men to permit sex-specific analyses and evaluation of potential effect modification by baseline function, frailty, chronic disease, and previous physical activity.

Future trials should also prespecify primary outcomes, perform prospective sample-size calculations, incorporate clinically meaningful thresholds, and include appropriate active comparison conditions. Active comparators are particularly important for determining whether observed changes are specific to dance or reflect the effects of structured physical activity more generally [30,42].

Where the research question concerns comparable effectiveness rather than superiority, equivalence should be evaluated using prospectively defined equivalence margins and appropriate equivalence-testing methods rather than inferred from conventional non-significant tests [27].

## 5. Conclusions

This study provides real-world cross-sectional evidence on established community-based senior dance participation among older women. The most consistent correlations with participation duration were observed for integrated functional performance, namely functional mobility, repeated chair-rise performance, and functional aerobic endurance. These correlations remained statistically significant after adjustment for current weekly attendance frequency and age and after correction for multiple testing. In contrast, evidence for correlations with mental well-being, handgrip strength, device-estimated skeletal muscle percentage, and flexibility was less robust; the WBI-5 correlation did not remain significant after FDR correction.

No SD versus SD+PA difference remained statistically significant after multiplicity correction under the present operational definition of additional regular physical activity. Exploratory comparisons with physically inactive younger women showed a mixed pattern and did not establish functional equivalence. Descriptively, both older senior dance groups had more favorable mean values than IOA across the measured outcomes, but the non-randomized groups differed substantially in baseline characteristics and total physical activity was incompletely characterized; these differences therefore cannot be attributed specifically to senior dance.

The findings describe cross-sectional associations and do not permit causal inference. Senior dance therefore warrants prospective evaluation as a multidomain community-based activity for older women, particularly in relation to integrated functional performance. Prospective longitudinal studies and adequately controlled trials are required to determine whether participation leads to sustained changes in functional performance, mental well-being, independence, and other clinically relevant outcomes.

## Figures and Tables

**Table 1:** Sociodemographic and health-related characteristics of study participants.

	SD (*n* = 66)	SD+PA (*n* = 34)	IOA (*n* = 30)	IYA (*n* = 30)
Age (years), mean (± standard deviation)	73.05 (±5.24)	70.44 (±5.33)	73.33 (±7.94)	33.2 (±8.42)
BMI (kg/m^2^), mean (±standard deviation)	26.73 (±4.59)	26.42 (±5.16)	26.43 (±5.01)	26.15 (±5.82)
Chronic diseases				
Cardiological disease, *n* (%)	10 (15)	9 (26)	6 (26)	3 (10)
Pulmonological disease, *n* (%)	7 (11)	7 (20)	5 (16)	0
Hypertension, *n* (%)	28 (42)	16 (47)	16 (53)	3 (10)
Lower limb arthritis, *n* (%)	14 (21)	9 (26)	7 (23)	3 (10)
Osteoporosis, *n* (%)	7 (11)	7 (20)	7 (23)	0
Persons with previous fall, *n* (%)	8 (12)	5 (14)	20 (66)	2 (7)

Note: Shading denotes category headings. BMI, body mass index; SD, senior dance only; SD+PA, senior dance plus additional regular physical activity; IOA, physically inactive older adults; IYA, physically inactive younger adults.

## Data Availability

Participant-level data are not publicly available because of ethical, consent, and institutional data-protection restrictions. Aggregated data supporting the findings are reported in the article. Further information regarding data availability may be obtained from the corresponding author upon reasonable request.

## Abbreviations

The following abbreviations are used in this manuscript:

2MST	2-Minute Step Test
ANOVA	Analysis of variance
BH	Benjamini–Hochberg
BIA	Bioelectrical impedance analysis
BMI	Body mass index
CI	Confidence interval
FDR	False discovery rate
IOA	Physically inactive older adults
IPAQ-SF	International Physical Activity Questionnaire–Short Form
IYA	Physically inactive younger adults
PA	Physical activity
SD	Senior dance only
SD+PA	Senior dance plus additional regular physical activity
STROBE	Strengthening the Reporting of Observational Studies in Epidemiology
TUG	Timed Up and Go
WBI-5	Hungarian four-response Five-Item Well-Being Index

## Appendix A. Graphical Summary of Study-Group Classification

Figure A1 provides a visual summary of the operational criteria used to classify participants into the four study groups. The figure is intended as a graphical aid to the definitions reported in Section 2.4 and does not introduce additional eligibility or exposure criteria.
